# Effects of body mass index on the outcomes of percutaneous nephrolithotomy

**DOI:** 10.1590/S1677-5538.IBJU.2016.0678

**Published:** 2017

**Authors:** Cemal Selcuk Isoglu, Tufan Suelozgen, Hayal Boyacioglu, Gokhan Koc

**Affiliations:** 1 Department of Urology, Tepecik Education and Research Hospital, Izmir, Turkey;; 2 Department of Statistics, Ege University Faculty of Science, Izmir, Turkey

**Keywords:** Body Mass Index, Nephrostomy, Percutaneous, Calculi

## Abstract

**Objective:**

To examine the the effect of body mass index (BMI) on PNL results and complications with a large number of patients.

**Materials and Methods:**

A total of 958 patients were included in the study, who underwent percutaneous nephrolithotomy in our clinic between 2008 and 2015. Patients were divided into 2 groups according to their body mass index. Patients with a BMI < 30 kg/m^2^ were classified as group 1 (n:676) and patients with a BMI ≥ 30 kg/m^2^ were classified as group 2 (n:282). Achieving stone-free status or having residual stones of ≤ 4 mm were considered as operational success.

**Results:**

The mean age was 47.9 years for group 1 and 48.9 years for group 2 patients. At postoperative first month CT analysis, residual stone was not observed in 466 patients (69%) of group 1 and 20 (72%) patients of group 2. There was no significant difference between the groups in terms of stone-free status (p=0.348). There was no significant difference between two groups complications. Also, there was no difference between the groups for requiring additional intervention (p=0.924). No other complications were observed in the patients.

**Conclusions:**

BMI does not affect the outcomes of percutaneous nephrolithotomy as well as complication rate.

## INTRODUCTION

Obesity is a widespread health problem with an increasing frequency all over the World, due to inadequate diet and decreased physical activity (1). It is well known that urinary tract stone disease is more common in obese patients (2). Percutaneous nephrolithotomy (PNL) is accepted as the gold standard treatment method for renal stones greater than 2 cm with high stone-free and reasonable complication rates (3). However, the results of PNL in obese patients are controversial. There are studies in the literature, indicating that obesity negatively influences the outcome of the operation as well as studies stating that it has no effect (4, 5). In this study, we investigated the effect of body mass index (BMI) on PNL results and complications.

## MATERIALS AND METHODS

We reviewed the data of 1150 patients who underwent PNL in our clinic between 2008 and 2015, retrospectively. Patients under the age of 18, who had an urinary system anomaly and underwent open stone surgery at the operation side were excluded from the study. A total of 958 patients with complete demographic, intraoperative, and postoperative data and having no exclusion criteria were included in the study. Patients were divided into two groups with respect to the body mass index. Those with a BMI < 30 kg/m^2^ were classified as non-obese (Group 1), whereas those with a BMI ≥ 30 kg/m^2^ were classified as obese (Group 2).

All patients were examined by non-contrast whole abdomen CT before the operation. In imaging studies, only the stones found in one calyx or only in the pelvis were named as simple stones while pelvic stones filling one calyx or more as well as staghorn stones were named as complicated stones (6).

The period, starting from the time of contrast agent administration to the patient at the prone position, up to the time of nephrostomy catheter insertion was recorded as the operation time. Duration of fluoroscopic imaging throughout the operation, the number of accesses, and the presence of intraoperative complications were also recorded. At the end of the operation, a 14 Fr re-entry Malecot catheter was placed in all patients and removed on postoperative day 1-3 and the patients without any complications were then discharged from the hospital. Furthermore, postoperative complications and additional interventions were noted for each patient. Complications were also classified according to Modified Clavien system.

All patients were reevaluated with non-contrast CT taken routinely at postoperative 1 month. The presence of residual fragments of ≤ 4 mm or no stone was considered as therapeutic success (7).

## SURGICAL PROCEDURE

Complete blood count, biochemical tests, coagulation tests and urine culture were performed in all patients preoperatively. Appropriate antibiotic treatment was given to the patients with positive urinary cultures and they were operated when they had sterile urine cultures. The patient was placed in the prone position after an open-ended 6-Fr ureter catheter was inserted through the urethra via cystoscopy while in the lithotomy position. The procedure was performed under general anesthesia. Following the injection of an opaque agent from the ureter catheter, an access needle was introduced into the renal collecting system via the appropriate calyx under fluoroscopic guidance. After placement of the guiding catheter, the Amplatz dilator set was used in order to create the tract, first with a 6 Fr dilator followed by a 28-30 Fr dilator, using the single step method. The same technique was used for the patients with complex stones, where a second access was required. The stones were broken by using a 24 Fr nephroscope and an ultrasonic lithotripter. A 14 Fr malecot re-entry catheter was routinely inserted after the operation was terminated.

## STATISTICAL ANALYSIS

The chi-square test was used to assess the categorical variables among the groups. The Mann-Whitney U test was used in order to compare the differences between the two independent groups. *P* value <0.05 was considered significant. IBM SPSS version 15.00 was used for analysis.

## RESULTS

There were 676 patients in Group 1 and 282 patients in Group 2. Of the patients, 422 were male (62.4%) and 254 were female (37.6%) in group 1, while 150 were male (53.1%) and 132 were female (46.9%) in group 2. The groups were similar in terms of gender (p=0.557).

The mean age of the patients was 47.9±14.7 years in group 1 and 48.9±13.3 years in group 2. There was no statistically significant difference in terms of age between the two groups although the patients in the obese group were slightly older than others (p=0.065).

Among Group 1 patients, 387 (57.2%) had simple stones while 289 (42.8%) had complex stones. In group 2, 156 (55.3%) had simple stones and 126 (44.7%) had complex stones. There was no statistically significant difference between two groups in terms of complexity and simplicity of the stones (p=0.583).

The mean duration time of operation was 72.4±3.81 minutes in the non-obese group while 65.4±2.75 minutes in the obese group. The mean duration of operation was not statistically significantly different between the two groups although it was relatively longer for group 1 patients (p=0.683).

The mean duration of fluoroscopy was 149.3±6.06 seconds in Group 1 patients and 144.4±9.74 seconds in group 2 patients, displaying no statistically significant difference between the two groups (p=0.803).

In the non-obese group, 580 patients had one, 83 had two, and 13 had three accesses. In the obese group however, 251 patients had one, 25 had two, and 6 had three accesses.Two groups were similar in terms of the number of accesses (p=0.311).

A total of 785 accesses were performed in 676 patients in Group 1. Of these, 709 (90.3%) were subcostal and 76 (9.7%) were intercostal access, through 11-12 intercostal space. In Group 2, totally 319 accesses were performed, of which 282 (88.4%) were subcostal and 37 (11.6%) were intercostal. There was no significant difference between the two groups in terms of the site of access (p=0.341) (Table-1).

**Table 1:** Significant preoperative and intraoperative data of the patients.

	Group 1	Group 2	P value

	(n:676)	(n:282)	
Mean age (years)	47.9	48.9	0.065
**Gender**			
Male	422 (62.4)	150 (53.1)	0.557
Female	254 (37.6)	132 (46.9)	
**Stone Load (%)**			
Simple	387 (57.2)	156 (55.3)	0.583
Complex	289 (42.8)	126 (44.7)	
Operation time (min.)	72.4	65.4	0.683
Fluoroscopy time (sec)	144.4	149.3	0.803
**Site of access (%)**			
Subcostal	709 (90.3)	282 (88.6)	0.341
Intercostal	76 (9.7)	37 (11.4)	
**Number of access**			
One	580	251	0.311
Two	83	25	
Three	13	6	
Stone-free status (%)	466 (69)	203 (72)	0.348

Due to intraoperative hemorrhage resulting in hypotension, blood transfusion was given to 12 patients (1.7%) in group 1 and 4 patients (1.4%) in group 2. No significant difference was detected between the two groups, in terms of intraoperative blood transfusion (p=0.695). Except for hemorrhage, no other intraoperative complication was observed in the patients.

Blood transfusion was required due to postoperative hemoglobine loss and hemodynamic instability such as hypotension in 24 (3.5%) patients in group 1 and 7 patients (2.4%) in group 2. Postoperative transfusion was grade 2 according to the Modified Clavien Classification System and showed no difference between the groups (p=0.395). No patient needed more than 2 units of transfusion.

Fever (>38ºC) was determined before discharge in 27 patients of the obese group and 70 patients of the non-obese group, and they were appropriately treated before being discharged from the hospital. No significant difference was detected in terms of postoperative fever between the groups (p=0.715). None of the patients developed sepsis or died from operation-related complications. Fever was considered as grade 1 complication group according to the Modified Clavien Classification System.

When the groups were evaluated in terms of operation success, postoperative residual stone fragments were observed in 210 patients (31%) of group 1 and in 79 patients (28%) of group 2. There was no significant difference in operative performance between the groups (p=0.348).

The groups were also evaluated for additional interventions. Thirty-six patients of the non-obese group required additional interventions after their discharge from the hospital. Double-J stents (DJS) were inserted in twenty-six patients due to wound site discharge or severe colic pain. Ureteroscopy (URS) was performed in twenty-six patients for treatment of the ipsilateral ureteral stones determined by non-contrast abdominal CT. In group 2 however, a total of 15 patients required additional interventions. Eight patients were inserted DJS because of the wound site discharge. Six patients underwent URS due to ureteral stone. Due to postoperative persistent hematuria in one patient, selective angiography was applied and upon determination of arteriovenous fistula, eventually superselective embolisation was performed. No additional treatment was required after embolisation. The requirement for additional intervention was considered as Clavien grade 3 and there was no significant difference between two groups (p=0.924) (Table-2).

**Table 2:** Intraoperative and postoperative complications of the groups.

	Group 1	Group 2	p value

	(n:676)	(n:282)	
Intraoperative transfusion (%)	12 ( 1.7)	4 ( 1.4)	0.695
Postoperative transfusion (%)	24( 3.5)	7 (2.4)	0.395
Postoperative fever (%)	70 (10.3)	27 (9.5)	0.715
**Additional interventions**			
URS	26	8	0.924
DJS	10	6	
Embolization	-	1	
**Modified Clavien Classification System**			
Grade 1	70	27	0.715
Grade 2	24	7	0.395
Grade 3	36	15	0.924

## DISCUSSION

Obesity has become a major problem for both developed and developing countries as a result of reduced physical activity and increased calorie intake. Particularly high income countries display higher rates of increase in obesity in the last three decades (1). The incidence of health problems such as metabolic syndrome, cardiovascular disease, malignancy, and renal calculus also increased in the community with the increase in obesity (8).

Today, PNL is a widely used method in renal stone treatment in both obese and non-obese patients. However, anestesia-related problems can be seen in obese patients. Respiratory complications may occur, for example a decrease in total lung capacity, as a result of the prone position during operation. Again, entubation difficulties may also occur in obese patients (9). In some centers, PNL is performed in the supine position in order to minimize such complications (10). In our clinic however, PNL in the supine position is not performed due to the lack of experience, yet complications related to anesthesia have not been observed.

In obese patients, it may not be possible to reach the renal collecting system with Amplatz sheet or it may be difficult to reach the stone because of the thick subcutaneous fat tissue. Curtis et al. reported that they made an incision in the skin and adipose tissue and retracted the tissue and so gained extra distance (11). In our study, standard Amplatz dilatators were used in both groups and access was obtained in all patients.

Carson et al. compared 44 obese and 226 non-obese patients and found no significant difference between the groups in terms of operation time, stone-free status and complication rates (12). In their study conducted with 236 patients (57 obese, 279 non-obese), Pearle et al. found no significant difference between the groups in terms of operative success and complication rates whereas longer operation time and higher rates of blood transfusion in the obese group (13). El-Assmy et al. indicated the mean stone size as 2.5±0.85 cm and reported no correlation between BMI and operative success (14). Alyami et al. examined patients having renal stones smaller than 3 cm and determined no correlation between BMI and operative success (5).

Croes study group published the first prospective study including 3709 patients (15). Patients with PNL were sorted according to their BMI. In contrast to many publications, they indicated a decrease in the rate of stone-free status whereas an increase in the duration of the operation in parallel with the increased BMI. In a series of 530 patients, Fearber et al. determined significantly higher complication rates in obese patients than in patients with normal BMI (16). Meanwhile, Pearle et al. reported that the need for blood transfusion was higher in the group with obese patients (13). Koo et al. examined a series of 181 patients in 4 groups and determined no difference between them in terms of operation time and blood loss (4).

In our study with 958 patients (282 obese and 676 non-obese), no significant difference was determined between two groups in terms of operation time, duration of fluoroscopy, operational success, need for additional intervention and complication rates. Even when the complications were grouped according to Clavien classification, both groups showed no significant difference. In the obese group, arteriovenous fistula developed in one patient and it was successfully treated with selective embolization. None of the patients died due to complications.

As for the limitations of the study, we can mention its retrospective design and inability to group the Clavien 3 classification into 3A and 3B because of the missing information about if the patients with Double J stent were given anesthesia or not.

## CONCLUSIONS

According to the results of our study, PNL is an effective method with high success rates, therefore it can be applied safely in obese patients as well as in non-obese patients.
